# Nano assembly of NiFe spheres anchored on *f*-MWCNT for electrocatalytic reduction and sensing of nitrofurantoin in biological samples

**DOI:** 10.1038/s41598-020-69125-5

**Published:** 2020-07-23

**Authors:** Kuo-Yuan Hwa, Tata Sanjay Kanna Sharma

**Affiliations:** 1 Graduate Institute of Organic and Polymeric Materials, National Taipei University of Technology, Taipei, Taiwan, ROC; 2 Department of Molecular Science and Engineering, National Taipei University of Technology, Taipei, Taiwan, ROC; 3 Center for Biomedical Industry, National Taipei University of Technology, Taipei, Taiwan, ROC

**Keywords:** Electrochemistry, Synthesis of graphene

## Abstract

The current study reports a facile simple, low-cost electrochemical sensor in the detection of nitrofurantoin (NFT) by using NiFe/*f*-MWCNT hybrid composite as a promising electrocatalyst. NFT is an antibiotic drug that is extensively using in pharmaceuticals and also in animal food production which causes a severe threat for both human and animal environments. Extending the residues of NFT are left into rivers, soils, lakes, and groundwaters either found or discharged leading health issues. To this NiFe/*f*-MWCNT composite was synthesized using a hydrothermal mechanism and then ultrasonicated to form a hybrid composite for catalytic evaluation and electrochemical detection of NFT for the very first time. Furthermore, the physicochemical properties of NiFe nanospheres conjugated on *f*-MWCNT are scrutinized using various analytical and spectroscopical techniques. Resulting transmission electron microscopy (TEM) displays a chain like NiFe nanospheres anchored on *f*-MWCNT with a well-defined spherical shape, without any comprehensive agglomeration. The NiFe*/f*-MWCNT screen printed carbon paste electrode (SPCE) displayed an excellent electrocatalytic activity for NFT with a LOD of 0.03 µM and a sensitivity of 11.45 µA µM^−1^ cm^−2^. establishing a new selectivity and with the existence of co-interfering compounds. To enhance the practical abilities analysis were performed in Human serum and urine samples which resulted in satisfactory recoveries with high precision and linear accuracy illustrated in Scheme 1.

## Introduction

Nitrofurantoin [(NFT)-*N*-(5-nitro-2-furfurylidene)-1-aminohydantoin], a well-known antimicrobial drug that has shown to actively inhibit several gram-positive as well as gram-negative microbes which belong to the family of nitrofuran^1,2^. This drug also has great importance in veterinary medicine in the prevention and treatment of coccidiosis that occurs in poultry and livestock^3,4^. It has also been associated with patients of neuropathy, hepatitis, pulmonary fibrosis, and hemolytic anemia suffering from the deficiency of glucose-6-phosphate^5^. Despite having such excellent antimicrobial activity, NFT showed some amount of metabolic toxicity^6^. The antimicrobial activity of NFT might owe to the fact that 5-nitro function undergoes reductive metabolic activation to form hydroxylamine derivatives, nitroso derivatives, and anion radical which includes a reduction mechanism of the nitro group (–NO_2_)^7,8^. Basically, the nitrofuran family consists of furaltadone, furazolidone, difurazone, nifurtoniol, nitrofurazone, nifuroxazide, and nitrofurantoin. Conspicuously, within a single daily oral dose usage, NFT gets partially metabolized where 25–50% is excreted as its original state with 1–2% excretes through urine in the form of aminofurantoin^9^. NFT also has the versatile ability in generating reactive species of oxygen and NO through its nitro group reduction. Where, by all these factors NFT is vividly released into environmental habitats making the contamination in the ecosystem, even at a quite low amount of level it is harmful and dangerous^10^. So, to overcome the exposure monitoring of NFT in the environment, food and pharmaceutical levels are necessary for avoiding risk which is associated. It is a necessity to develop a highly sensitive, accurate and rapid real-time analytical tool to detect the presence of NFT^11^. In pursuit of this goal, several methods have already been reported for the determination of NFT such as colorimetry, spectrophotometry, polarography, high-performance liquid chromatography (HPLC), and reductive flow-injection amperometry^12^. However, these methods did not provide a satisfactory quantification limit for detecting the drug. Also, these methods required pre-treatment of the samples that was a time-consuming process. But, the electrochemical methods had several advantages such as cost-effectiveness, high selectivity and sensitivity, rapid performance, and portability to which they are considered as cutting-edge technology^13^. It was revealed from the literature survey that till now only a handful of electrocatalysts have been reported for the precise detection of NFT due to its remarkable advantages.

The development of electrochemical sensors using carbon sources, nanostructured metals, metal oxides, and their nanocomposites have gained a significant research interest due to their excellent optical, electronic, and chemical properties^14–17^. The synthesis of metal nanoparticles, especially bimetallic alloys has gained the extensive interest of the researchers as they possess properties like high conductivity, chemical stability, and exhibit good electrocatalytic activity^18^. The bimetallic alloys have been widely used for various electrochemical applications such as energy storage, sensing platforms, and energy conversion^19^. The most commonly used metals for electrocatalytic activities are Au, Pt, Ag, and Pd due to their intrinsic physiochemical properties^20^. Although these metals are excellent electrocatalysts, yet their expensiveness makes them inappropriate for real-time applications^21–23^. In order to overcome these drawbacks, the 3d-transition metals such as Mn, Fe, Cu, Ni, Zn, and Co are being explored as an electrocatalyst as these metals are abundant and non-toxic in nature, as well as cost-effective enriched with electron capacity^24^.

**Scheme 1:**
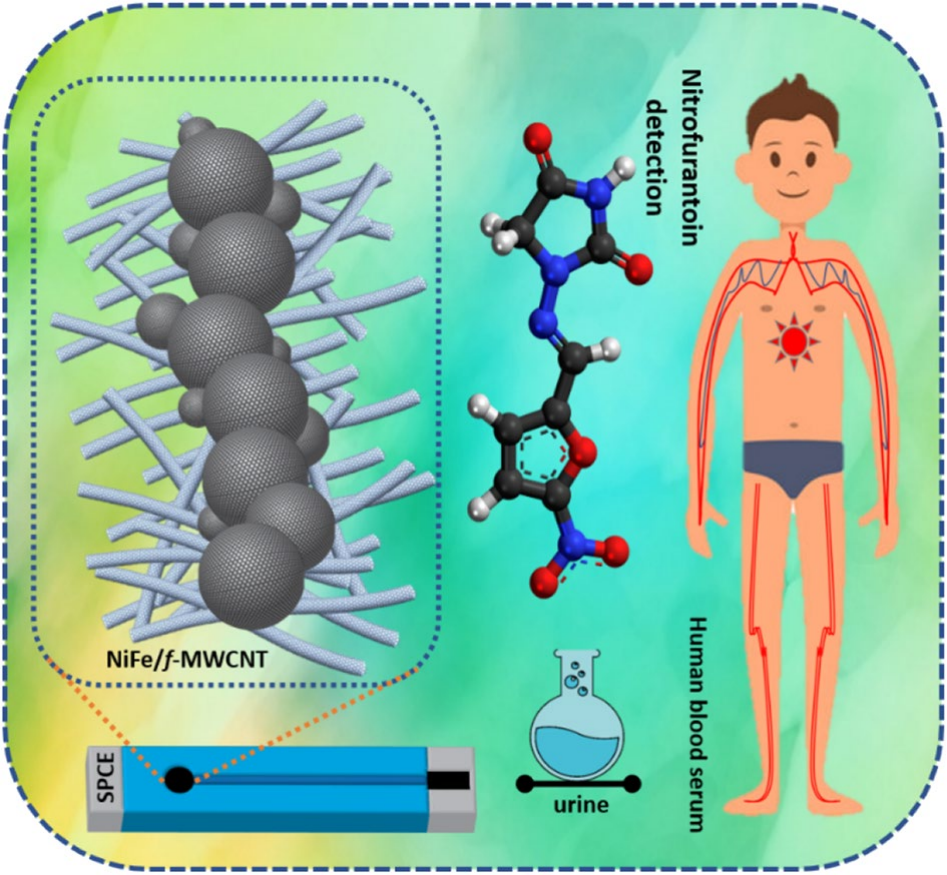
Abstract illustration of NiFe anchored on *f*-MWCNT as a hybrid composite for electrochemical detection of NFT biological samples.

On the other hand, carbon materials like carbon nanofiber (CNF), graphene, graphitic carbon nitride, and Multi wall-carbon nanotube (MWCNT)^25^ has considered a high impact over the decades for researchers in analytical electrochemistry in developing a high-performance material towards electrode in co-ordinance of electrochemical applications^26,27^. Whereas, MWCNTs are fiber-like structured which is a carbon nanomaterial that has a peculiar electronic property with the diverging mechanical property; which is being used as an extrinsic electrochemical applicative. MWCNTs are extremely active in edge sites of the outer walls were comparatively less in order due to the enrichment in the electron transferability^28,29^. Although pure MWCNTs are poor in sensing performance, as well as an aqueous dispersion. To overcome the issue and to enhance the electrocatalytic activity of MWCNTs are functionalized (-COOH) or condensed by polymers with organic molecules^30^. Moreover, the functionalization of MWCNTs (*f*-MWCNT) invokes a complex procedure with multiple reactions by using toxic acids^31^ (Scheme 1).

**Scheme 2:**
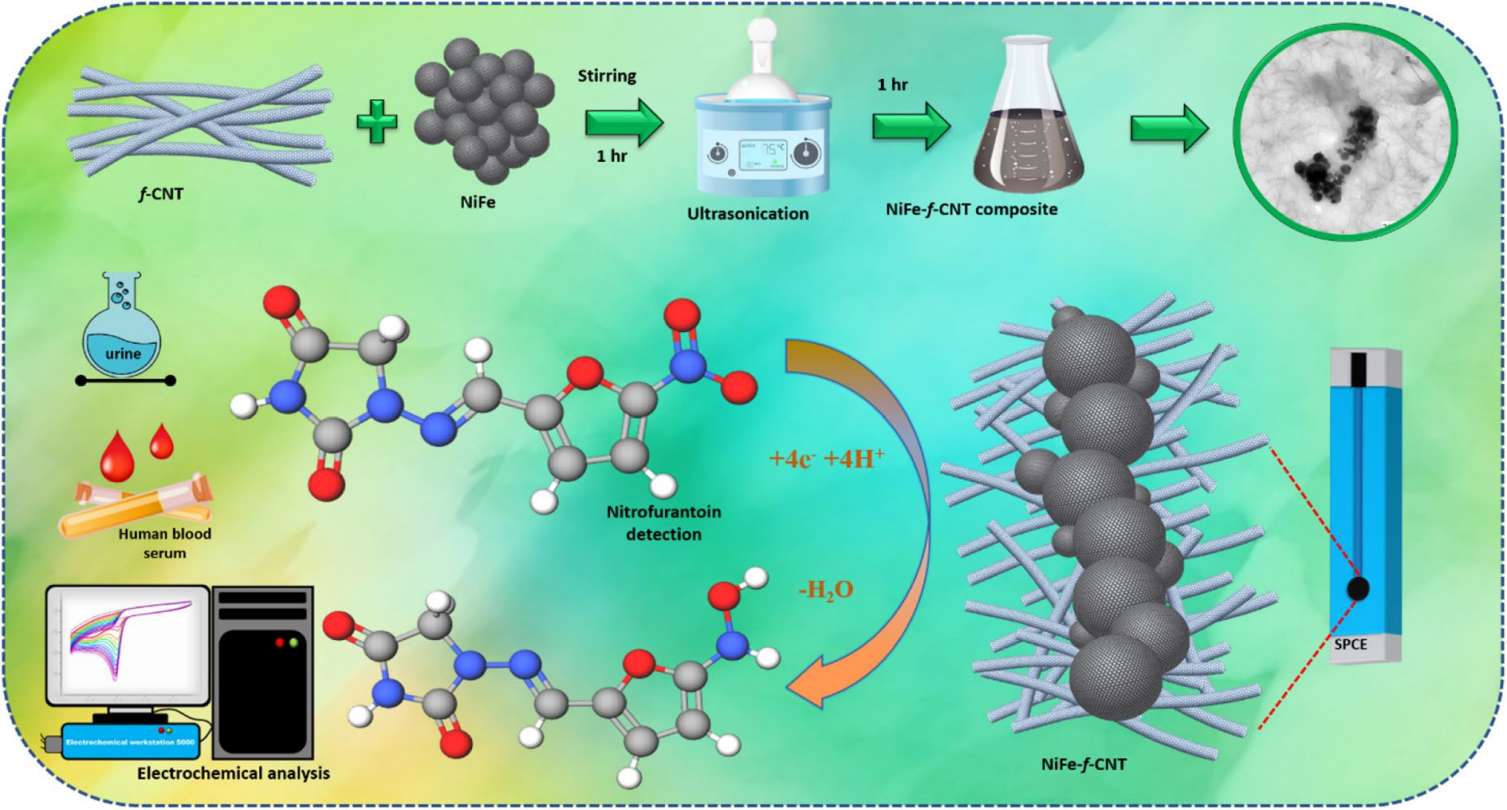
Schematic illustration of NiFe/*f*-MWCNT hybrid composite with electrochemical detection in NFT.

Therefore, developing an eco-friendly method to design a sensor that delivers an outstanding electrochemical performance is highly desirable. Huge efforts have been put by the researchers to develop an electrochemical sensor that is highly robust, precise, and at the same time cost-effective to be used for practical applications. To this, we have prepared a NiFe/*f*-MWCNT hybrid composite using a facile and simple one-pot hydrothermal reaction in preparation of NiFe nanospheres anchored on *f*-MWCNTs for sensing of urinary anti-microbial drug NFT using screen printed carbon paste electrode (SPCE). To understand more about NiFe nanospheres anchored on *f*-MWCNTs a surface morphological studies are persuaded using TEM, FE-SEM, FT-IR, and XPS. The electrocatalytic analysis was done using NFT for bare electrode, modified NiFe/SPCE and composite NiFe/*f*-MWCNT/SPCE. Electrochemical analysis results demonstrating NiFe nanospheres anchored on *f*-MWCNTs with huge potential in designing an environment-friendly electrochemical sensor (Scheme 2).

## Results and discussion

**Figure 1:**
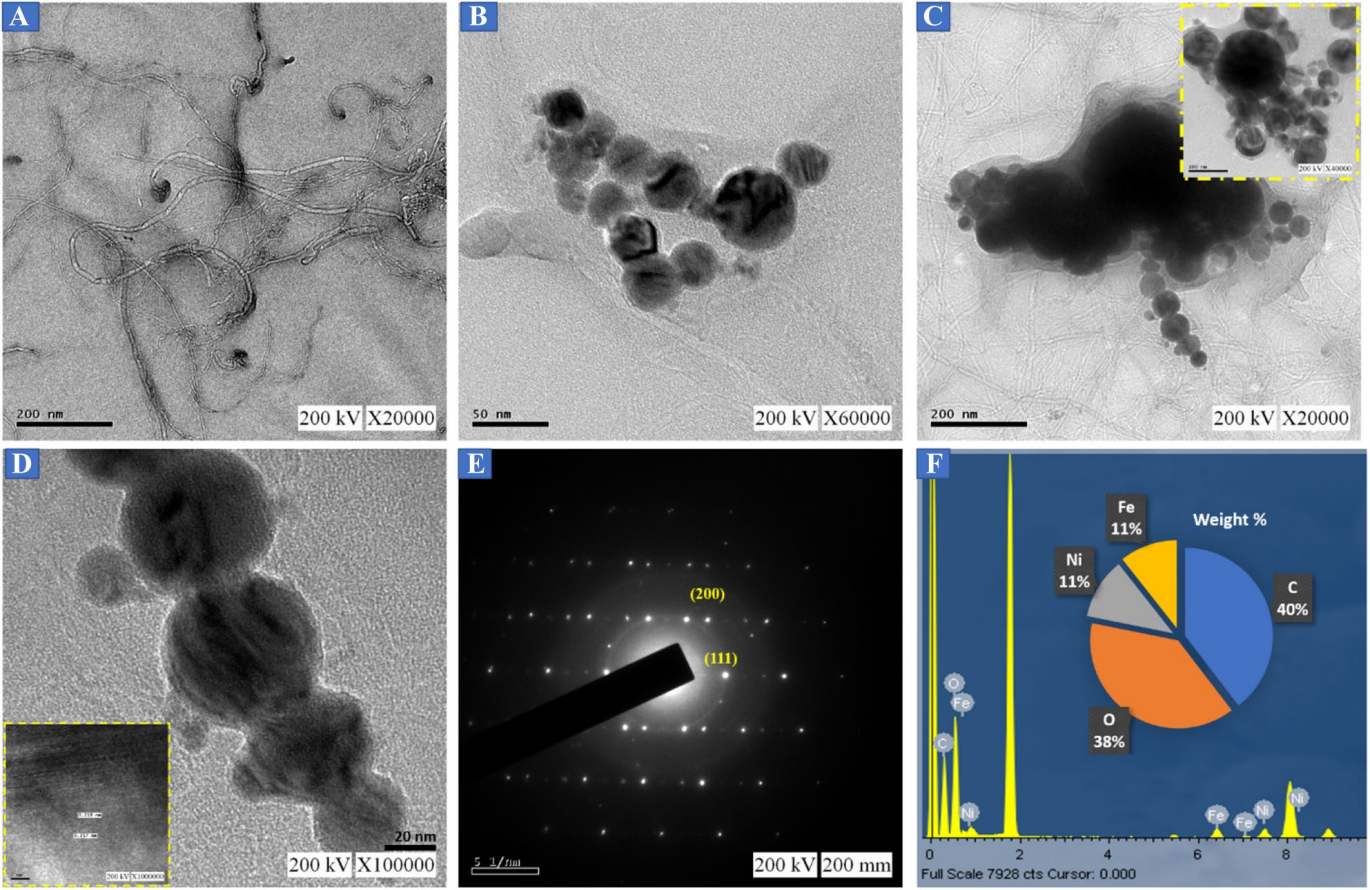
TEM images of (**A**) *f*-MWCNT, (**B**) NiFe, (**C**,**D**) NiFe-*f*-MWCNT composite, (**E**) diffraction pattern of NiFe-*f*-MWCNT composite, (**F**) EDX spectra of selected TEM image.

To investigate a comprehensive structural analysis on NiFe-*f*-MWCNT hybrid composite, TEM, EDX, XRD, XPS, and FT-IR has been carried out. In Fig. 1A. displays *f*-MWCNT an inhomogeneous mixture of distribution formed like a tubular structure. Figure 1B shows the NiFe Nano-spherical structure which is formed homogeneously and also in well-defined and well-structured with particle-sized. NiFe nanospheres are agglomerated in a systematic line phenomenon reveling the effect of ultra & probe sonication concerning period (Fig. S2). In Fig. 1C,D. exhibits NiFe-*f*-MWCNT composite under different magnifications, where a low characteristic magnification is exhibited and expanded till high magnification. Inset of Fig. 1D displays the HRTEM image with a lattice border arranged in a considerable d-spacing of 0.217 and 0.218 nm which bear a resemblance to planes (111) and (200) correspondingly of XRD patterns to NiFe nanospheres. In Fig. 1E selective area electron diffraction (SAED) pattern is exhibited in reliable to the XRD and HRTEM analysis obtained.

**Figure 2:**
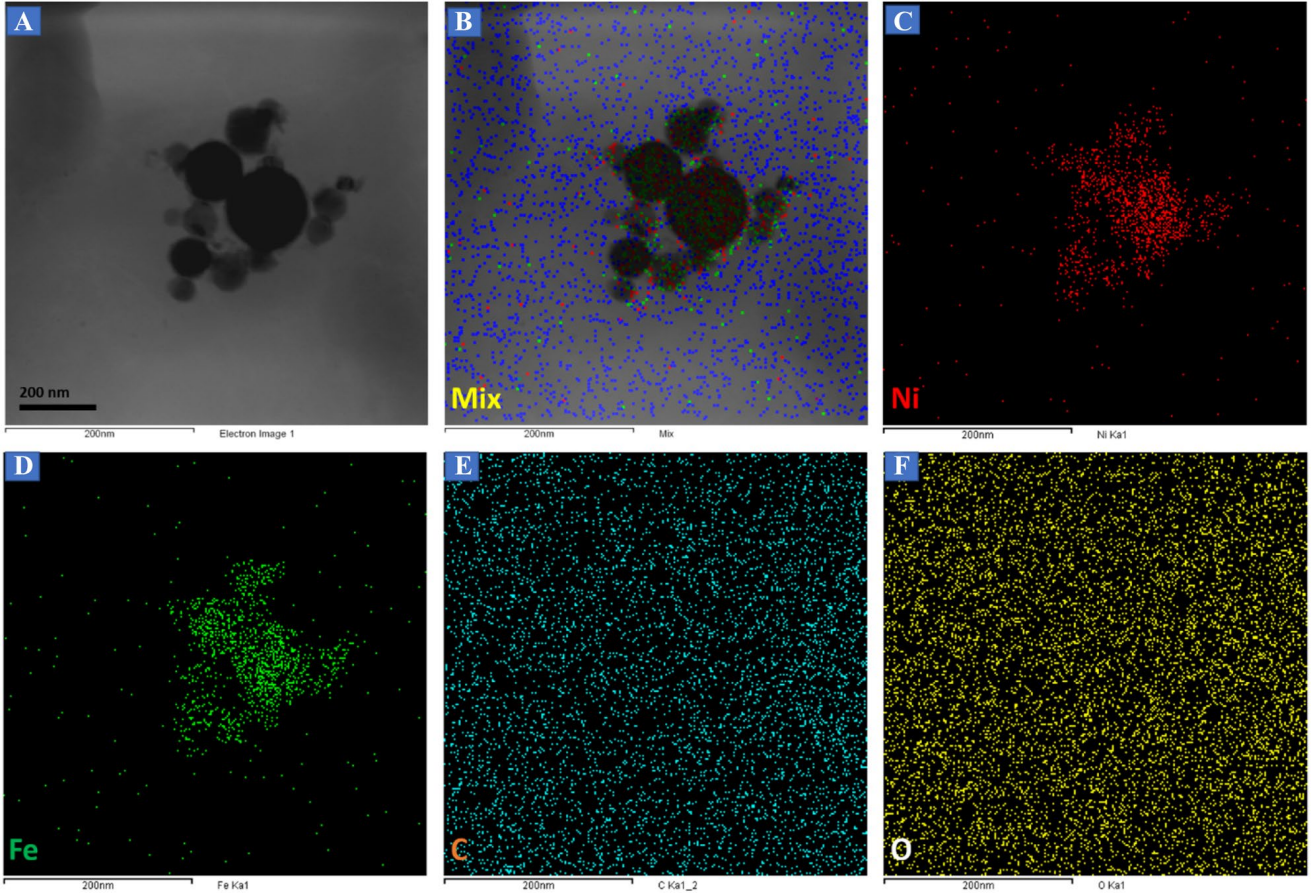
Elemental mapping of (**A**) NiFe-*f*-MWCNT composite, (**B**) mix, (**C**) Ni, (**D**) Fe, (**E**) C and (**F**) O.

Figure 1F displays the EDS mapping representing the elemental composition in the NiFe-*f*-MWCNT hybrid composite where the presence is predicted to be Nickel 11.22%, iron 10.63%, carbon 39.58% and oxygen 38.57% through which it is evident that the Ni, Fe, and C atoms have equally dispersed without any other interfering elemental traces of precursors^11^. Corresponding FE-SEM images are illustrated in Fig. S2. exhibiting *f*-MWCNT, chain-like agglomerate NiFe nanospheres, and NiFe-*f*-MWCNT hybrid composite. To confirm the presence of the elements elemental mapping is illustrated in Fig. 2 where Fig. 2A shows the agglomerated NiFe on *f*-MWCNT and its mixed combination in Fig. 2B,C Ni percentage and in Fig. 2D Fe percentage and in Fig. 2E carbon weightage and at last in Fig. 2F oxygen weight composition, which resembles that asper-prepared NiFe/ *f*-MWCNT has all the elements successfully. Interestingly, the line spectrum weight percentage is also illustrated showing the spectra positions of Ni, Fe, C and O in Fig. S3.

**Figure 3:**
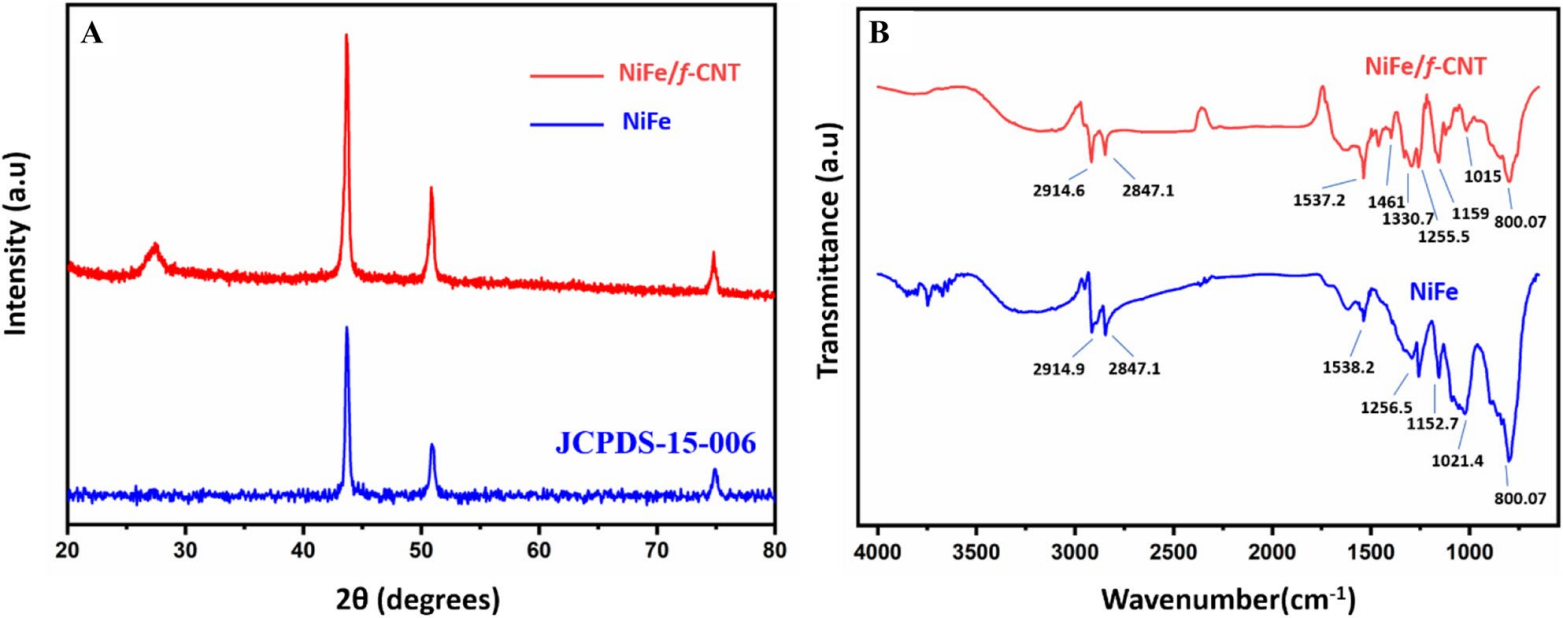
(**A**) XRD pattern of NiFe (blue) and NiFe-*f*-MWCNT composite (red). (**B**) The FT-IR spectrum of NiFe (blue) and NiFe-*f*-MWCNT composite (red).

Furthermore, to understand the crystallographic structure and as prepared samples phase purity is characterized using XRD measurement analysis. Figure 3A displays the XRD patterns of raw NiFe (blue) where diffraction peaks seemed to be at 43.2^ο^, 51.1^ο^, 74.9^ο^ in respect to (111), (200), and (220) with the cubic crystal plane structure and NiFe-*f*-MWCNT composite (red) with the peak diffractions at 27.1^ο^ (002), 43.4^ο^, 51.3^ο^, and 74.5^ο^, which exhibits that the composite is in fcc phase (JCPDS-15-006)^32–34^. Moreover, to understand the chemical structure and bonding nature between *f*-MWCNT and NiFe FT-IR spectroscopy analysis were carried out which is displayed in Fig. 3B. Here Blue spectra indicate NiFe and red indicate NiFe/*f*-MWCNT composite. Observing the NiFe/*f*-MWCNT spectra it is evident that multiple peaks are obtained at different bands ranging from 800 to 1538 cm^−1^ and 2,800 to 3,000 cm^−1^ this is possibly due to the –OH groups whereas the 1537 cm^−1^ is represented to be the characteristic C=O stretching vibration^35,36^. The peaks appeared at 1,152.7, 1,330.7 and 1,461 cm^−1^ are ascribed as C–OH stretching as it has the presence of hydroxyl and carboxyl group bounded to the basal planar region of*f*-MWCNT^37^. a sharp peak is observed at 800.07 cm^−1^ which can be attributed to bending vibrations of metal-sulfides. Therefore, results state that NiFe/*f*-MWCNT peak intensities are decreased when compared with NiFe which confirms a successful formation of NiFe on *f*-MWCNT.

**Figure 4:**
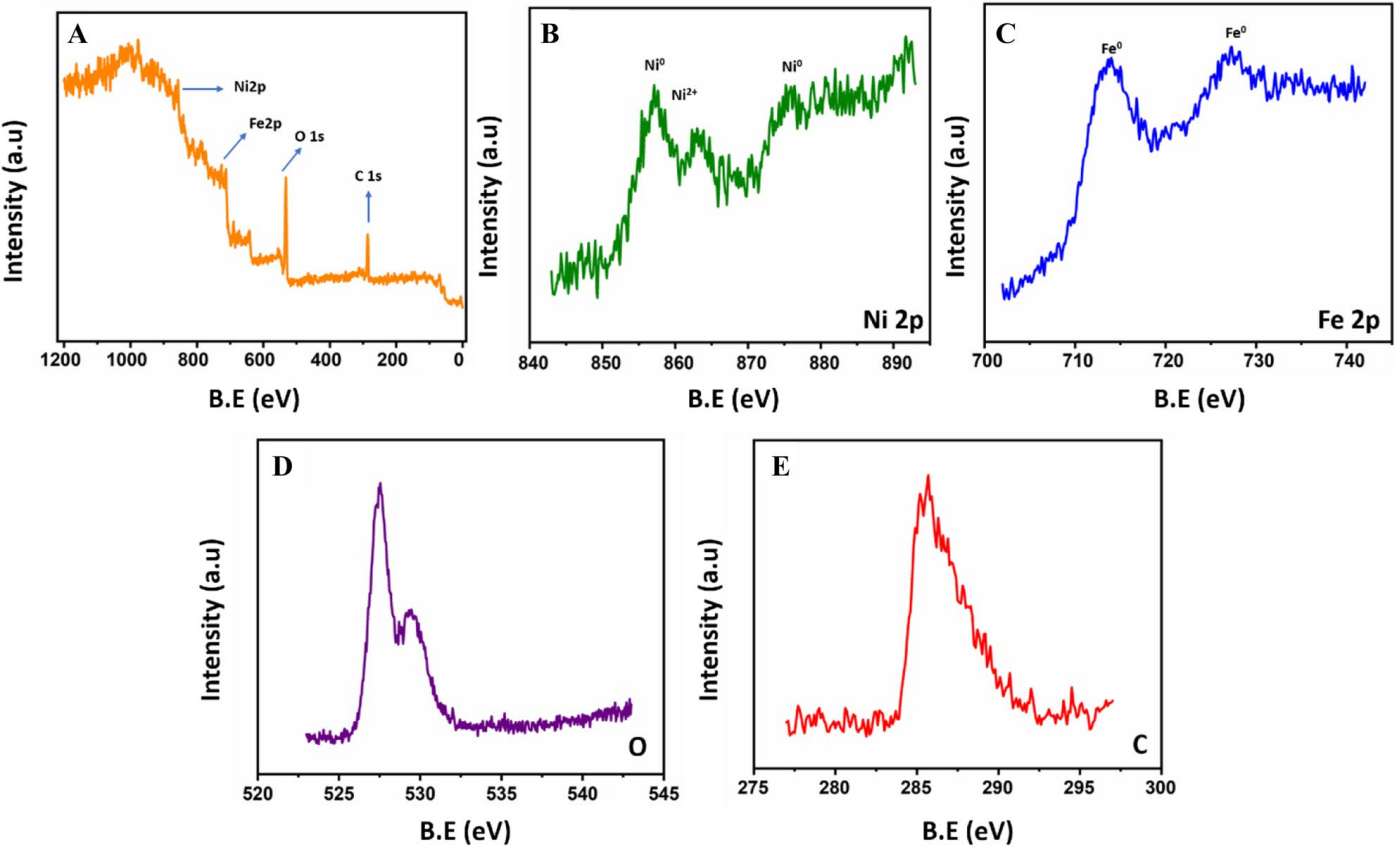
(**A**) XPS survey scan of NiFe/*f*-MWCNT, (**B**) C1s, (**C**) O1s, (**D**) Ni 2p, (**E**) Fe 2P nanospheres.

To enhance and confirm elemental composition XPS analysis is carried out for the NiFe-*f*-MWCNT composite. Where, Fig. 4A displays the XPS survey scan of NiFe-*f*-MWCNT which indicates the existence of Ni, Fe, and C in the prepared composite. Distinguished peaks are obtained in the XPS spectrum with 856.14 (2p_3/2_) and 876.2 eV (2p_1/2_) of Ni 2p which is displayed in Fig. 4B. This is perceptibly allotted for metallic Ni (neutral), In trace amount level a peak is visible at 857.6 eV which indicated that the presence of Ni^2+^ which is partially oxidized^38,39^. Whereas, two intense broad peaks are observed with a binding energy of 714.1 (2p_3/2_) and 726 eV (2p_1/2_) in the XPS spectrum of Fe 2p which is displayed in Fig. 4C through this it is attributing zero-valent iron existence^40,41^. Figure 4D displays a sharp peak which is obtained for the oxygen XPS spectrum at 527.2 eV. Figure 4E displays a broad peak attained for the carbon XPS spectrum at 285.1 eV^42^. Thus, the XPS analysis demonstrates the successful formation of bimetallic NiFe conjugation with f-MWCNT is zero valent with the presence of C, Ni, and Fe.

### Electrocatalytic analysis of NFT on NiFe/*f*-MWCNT modified hybrid electrode

**Figure 5:**
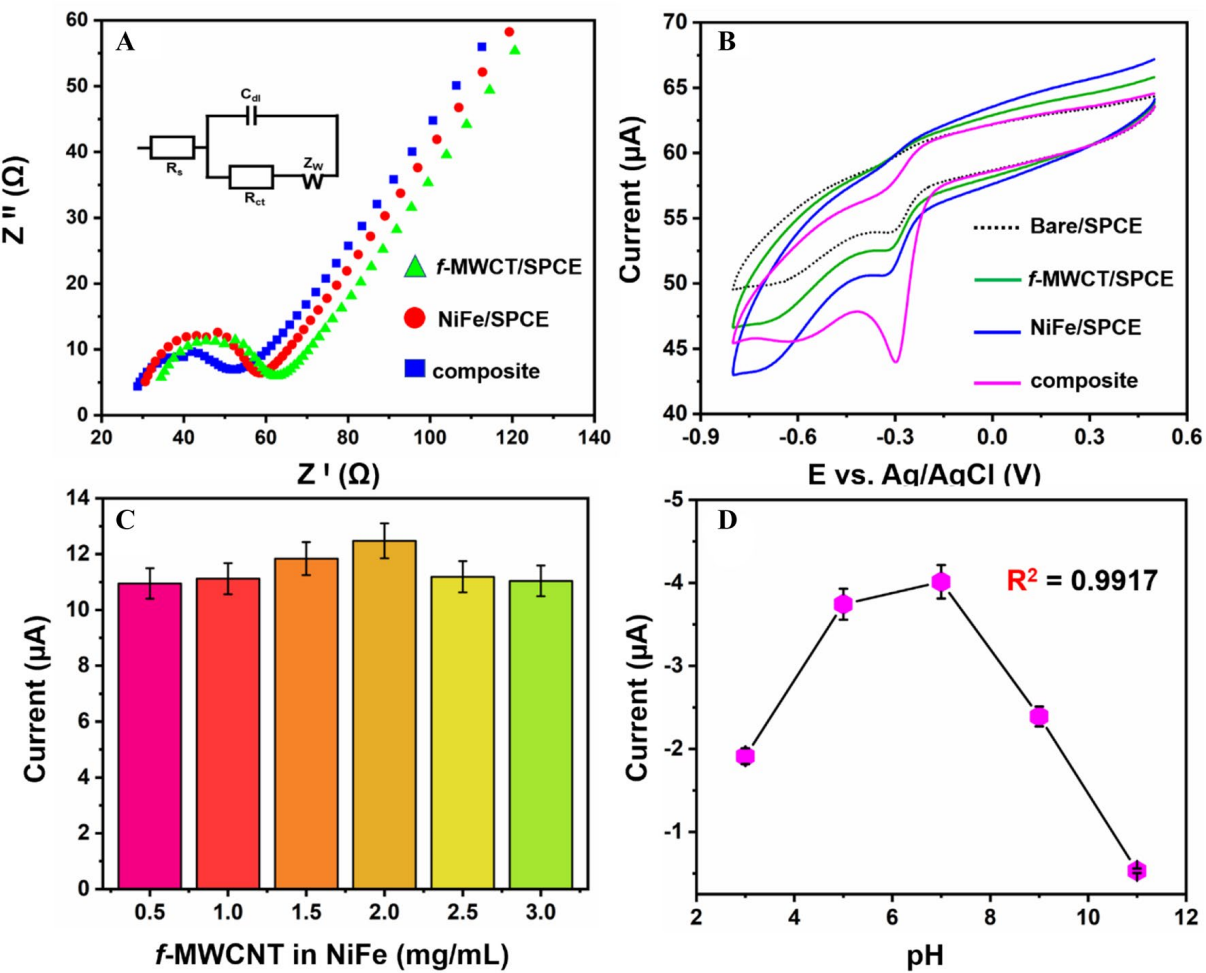
(**A**) EIS spectra of *f*-MWCNT, NiFe and NiFe/*f*-MWCNT hybrid composite in 0.005 mM of Fe(CN)_6_^3−/4−^ containing 0.1 M of KCl (inset: Randles equivalent circuit). (**B**) CV curves of bare SPCE, *f*-MWCNT/SPCE, NiFe/SPCE and NiFe/*f*-MWCNT/SPCE in the presence of 200 µM of NFT. (**C**) The effect of loading percentage of *f*-MWCNT in NiFe/*f*-MWCNT composite to a peak response of 200 µM of NFT. (**D**) Peak current response for different pH solutions.

Electrochemical impedance spectroscopy (EIS) is considered as an essential parameter to electrochemical sensors for its assessing in electrochemical sensing abilities and it can also distinguish about interfacial properties that occur between the as-per prepared electrode surface and its electrolytes. To verify the electron transportability of NiFe/*f*-MWCNT/SPCE hybrid composite was investigated using EIS, an existing of 0.005 M of Fe(CN)_6_^3−/4−^ in a 0.1 M of KCl. Figure 5A displays electrochemical impedance for different electrodes with its Nyquist plots explaining the phenomena of transferring abilities. Whereas, the linearity of the Nyquist plot at low frequency describes that a rapid electron transfer diffusion has occurred here the semi-circle corresponding for the limited electron transfer process. Continuing, charge transfer resistance (R_ct_) value of the modified electrode equally balances with the semi-circle diameter. In inset Fig. 5A displays the equivalent circuit model which involves the solution resistance (R_s_), double layer resistance (C_dl_), Warburg diffusion (R_w_), and also the charge transfer resistance (R_ct_). Fig. S4A displays bare SPCE which illustrates that a poor electron transfer has happened representing a large semicircular position. Although, when SPCE is modified with NiFe (curve a) and *f*-MWCNT (curve b) displayed an equivalent decrease when compared to the diameter of bare SPCE semicircle, illustrating *f*-MWCNT has an improved electron transfer process by the side of electrical conductivity^33^. Here, R_ct_ values of bare SPCE, NiFe, *f*-MWCNT and NiFe/*f*-MWCNT/SPCE are calculated as 132.56, 36.16, 28.12, 22.44 Ω individually. The lowest Rct value 22.44 for NiFe/*f*-MWCNT/SPCE describes that a high surface area and high electron transportability has happened for the surface of the NiFe/*f*-MWCNT proving that the hybrid composite has good conductivity, with diffusion for the redox probe of electrode interference. Thus, NiFe/*f*-MWCNT hybrid composite states a high electrochemical assessment for the detection of NFT.

### Electrochemical performance of NFT towards NiFe/*f*-MWCNT/SPCE

**Scheme 3:**
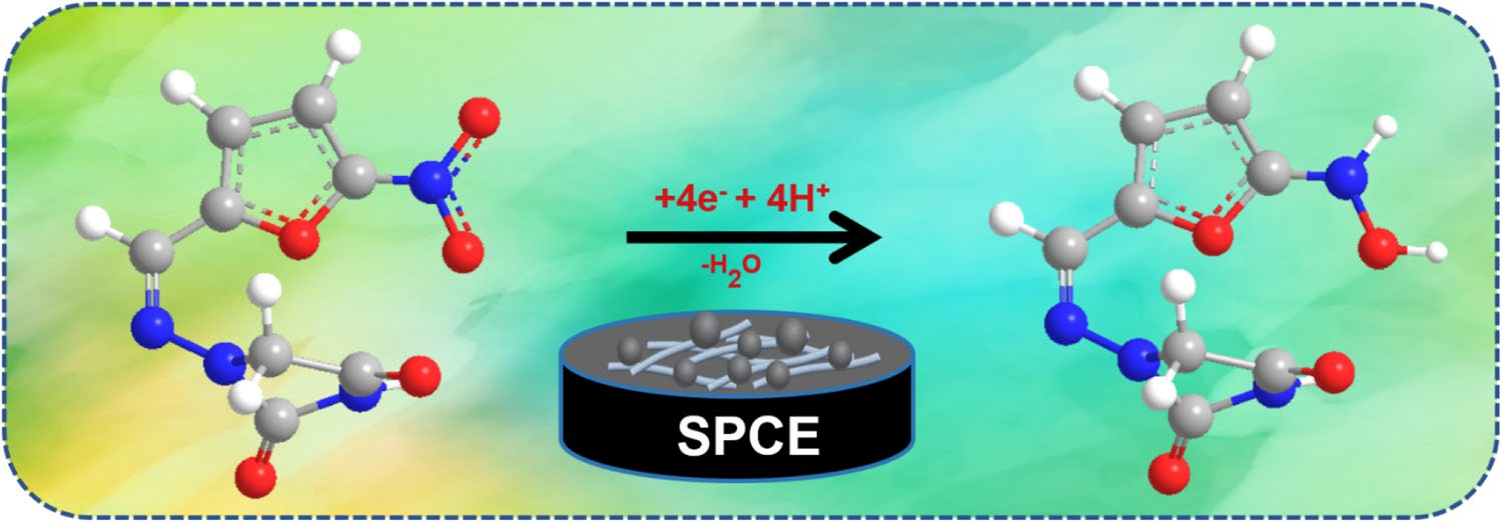
The electrochemical mechanism for NFT.

The electrochemical performance of NFT is investigated for bare SPCE, NiFe/SPCE and NiFe/*f*-MWCNT/SPCE using cyclic voltammetry at a pH of 7.0 in (PBS 0.05 M) in a constant scan rate of 50 mVs^−1^ within a potential range of − 0.9 to 0.6 V. The CVs of the modified electrodes are verified in the presence of 200 µM of NFT which is shown in Fig. 5B with an insignificant cathodic hump at I_pc_ = 3.43 µA which is observed at a peak potential of − 0.3 V (E_pc_) for bare SPCE (curve a), which is attributed to the nitro group of irreversible electroreduction in NFT. On bare SPCE NFT has displayed a reduction current emphasizing poor electron transfer rate capacity towards the electrode. Although, *f*-MWCNT has shown a slight variation in cathodic peak current of I_pc_ = 4.01 µA with a lover potential of E_pc_ = 3.16 V in curve b explaining the integration of *f*-MWCNT in NFT electrocatalytic reduction phenomena. Whereas the same comparison is taken with NiFe SPCE to analyze the electron transfer rate between *f*-MWCNT and bare SPCE to which in curve C a peak is observed with a hike in the cathodic peak in compared to other two curves showing an I_pc_ = 4.93 µA and potential E_pc_ = 3.21 V, proving that the NiFe has good transporting capacity in compared to other electrodes. Continuing, cyclic voltammogram analysis of NFT on NiFe/*f*-MWCNT/SPCE has conducted which is displayed in curve D exhibiting a definite, sharp peak current with good current I_pc_ response of 13.40 µA in a potential E_pc_ of 3.0 V. To NiFe/*f*-MWCNT/SPCE the reduction peak current which obtained is comparatively 1.47, 3.24, and 7.18-folds higher in surface to NiFe-SPCE, *f*-MWCNT-SPCE, and bare SPCE. Although, NiFe/*f*-MWCNT/SPCE electrode cathodic peak current foe NFT has shifted slightly − 95 mV in assessment towards bare SPCE. Over the potential enhancements of cathodic peak current response is minimized for NiFe-SPCE and *f*-MWCNT-SPCE which outclasses electrocatalytic performance of as per prepared electrodes in control comparing NiFe/*f*-MWCNT/SPCE electrode is constructed which has performed good electrocatalytic behavior this may be due to the rapid electron transfer rate, stabilized and improved electrical conductivity, with a surface to volume ratio^10^. where a possible mechanism explaining the process of NFT is shown in Scheme 3.

To enhance and understand the relationship between NiFe and *f*-MWCNT ratios and its electrochemical sense analysis on the loading ratio of NiFe (0.5, 1, 1.5, 2, 2.5, 3 mg mL^−1^) in preparing 1 mg mL^−1^ of NiFe solution is varied. Whereas the cyclic voltammograms as ratio varied composites are analyzed in 200 µM of NFT is displayed in bar representation Fig. S4B normalizing current response against the amount of NiFe ration loaded. By the results obtained it is noticed that cathodic peak current has increased when NiFe amount is increased within 2 mg mL^−1^, additionally when the amount of NiFe (> 2 mg) has added in the composite peak drop is observed in the current. Consequential to results a 2 mg mL^−1^ of NiFe solution mounted with 2 mg of *f*-MWCNT mL^−1^ is attributed as a perfect amount of ratio which is used in further electrochemical experiments. Usually in achieving good sensing performance thickness and size plays a vital role to overcome this factor catalyst loading analysis is carried out for SPCE surface in NFT electrocatalytic detection using CV studies. Here, different amounts of NiFe/*f*-MWCNT composite is loaded on to the surface of the SPCE (3, 4, 5, 6, 7, and 8 µL) for which 6 µL of NiFe/*f*-MWCNT distinguished a high peak current Fig. 5C. Subsequently, overloading or less loading of the composite on to SPCE surface resulted in hindering electron transfer rate between the analyte and surface of the electrode; less or more than 6 µL resulting with improper or less catalytic film response which is not evident in catalyzing electrochemical reduction on the surface SPCE electrode. Thus, 6 µL is considered as an optical amount to load on the surface of the electrode for enhancing and understanding the electrochemical studies of NFT.

### Effect of pH, concentration and scan rate for NFT

In cyclovoltammetry studies pH analysis is considered as one of the significant factors for developing an electrochemical sensor due to the dependence of peak current and potential on pH solution. Therefore, to analyze the variation effect in pH multiple pH’s are used like pH 3, 5, 7, 9 and 11 in CV for NiFe/*f*-MWCNT/SPCE electrode in detecting the reduction of 200 µM of NFT with a scan rate of 50 mV s^−1^. Whereas the differences between pH 3–11 are displayed in Fig. 5D with a difference in peak current and peak potential. Gradually peak current has increased which is evident by observing pH 3–7 and then a decrease in current can be noticed from pH 7–11 this is possibly due to the involvement of protons in the irreversible process of nitro-reduction in NFT solution^8^. Although the highest peak current is obtained in pH 7 to which obtained Epc has shifted to high potential when compared between pH 3–11, for which linear correlation coefficient value thus obtained is 0.9917. Using linear plot slope value is used in applying for Nernst equation Ep = − 0.0614 m/n pH + b in calculating proton and electro ratio m/n where the ratio is 0.71 matching previously reports in the detection of nitro compounds using electrochemical reduction mechanism. Thus, satisfying NFT has achieved by accompanying an equal number of protons and electrons in the electrochemical reduction process for the electrode reaction. Involving an electroreduction of –NO_2_ to– NHOH reaction towards NFT with 4H^+^ and 4e^−^ illustrated clearly in Scheme 3.

**Figure 6:**
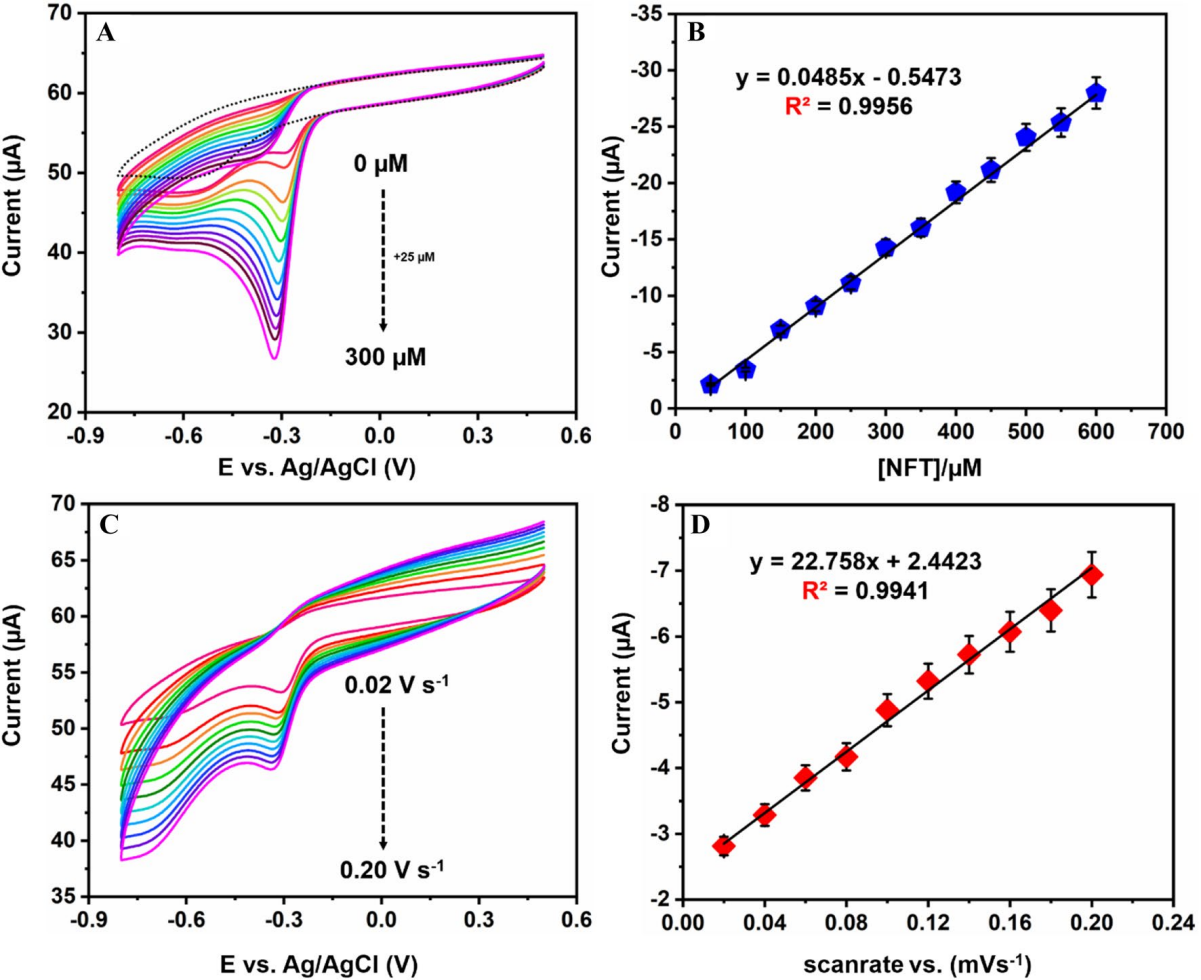
(**A**) CV response for NiFe/*f*-MWCNT/SPCE for different additions (0 to 300 µM) of NFT at a scan rate of 50 m V s^−1^. (**B**) calibration plot for different concentrations of NFT vs peak current response. (**C**) CV response of NiFe/*f*-MWCNT/SPCE for different scan rates ranging from (0.02 to 0.20 m V s^−1^) for 200 µM of NFT in pH 7.0. (**D**) Calibration plot for scan rate response vs peak current response.

Figure 6A illustrates CV response for NiFe/*f*-MWCNT/SPCE modified electrode by adding different amounts of concentrations of NFT in 0.05 M PBS solution pH 7.0 with a scan rate of 50 m V s^−1^. The results display that a sharp cathodic potential peak is observed for CV reduction of NFT with a potential peak at − 0.324 V. reduction peak current response for NFT to NiFe/*f*-MWCNT/SPCE modified electrode has gradually increased with the increase in the concentration of NFT from 0 to 250 µM for each addition 25 µM is added, signifying NFT in the proposed sensor. In comparison with bare SPCE as of absence of NFT, no peak is observed to NiFe/*f*-MWCNT/SPCE modified electrode. Through this, it is satisfied the proposed electrode has a good electrochemical response and electron transfer rate with the as per prepared composite in the reduction of NFT. Figure 6B illustrates cathodic peak current with a satisfying linear regression equation I_pc_ = 0.0485(NFT/µM)-0.5473 (R^2^ = 0.9956). thus, it represents a high electrochemical reduction for the enhancement of NFT in the as per prepared NiFe/*f*-MWCNT/SPCE electrode.

To understand and examine the kinetic reaction effect of the scan rate for the electroreduction of NFT was performed on NiFe/*f*-MWCNT/SPCE illustrated in Fig. 6C. Whereas the scan rate is performed by using CV through interchanging the sweep rates in lower to higher increasing order that is from 0.02 to 0.20 Vs^−1^, where the cathodic peak current has increased while increasing the sweep rates linearly. Due to the diffusion layer cathodic peak current is shifted towards the highest potential. Continuing, to the obtained scan rate kinetic cathodic peak current is plotted concerning the square root of the scan rate displaying a linear regression relation with a sequential correlation coefficient of 0.9941 illustrated in Fig. 6D. Demonstrating, the absorption of NFT on NiFe/*f*-MWCNT/SPCE surface has undergone a diffusion-controllable mechanism through irreversible phenomenon.

### Calibration curve of NFT

**Figure 7:**
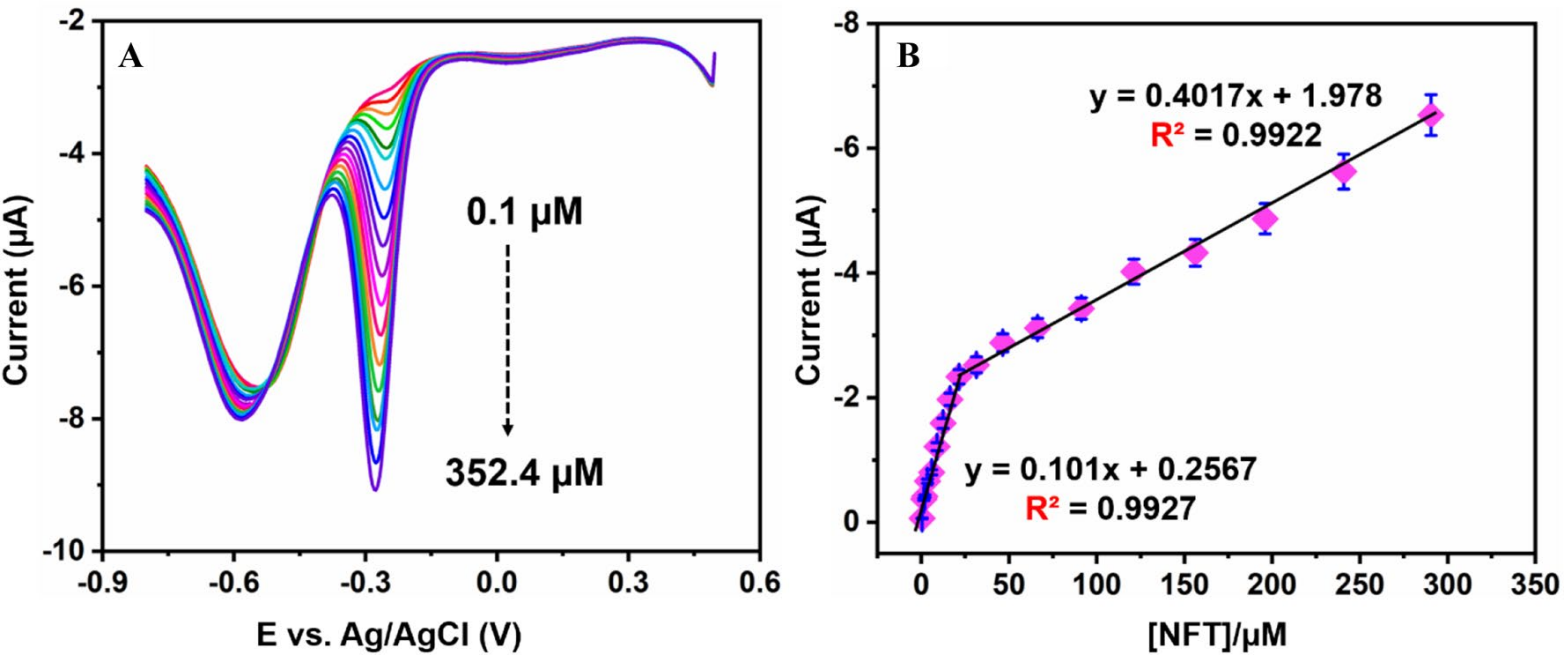
(**A**) DPV current response for different concentrations of NFT in pH 7.0 for NiFe/*f*-MWCNT/SPCE electrode. (**B**) calibration plot for different concentrations of NFT acquired from DPV vs response current (n = 3).

Differential pulse voltammetry (DPV) is considered as one of the finest methods to detect due to its high sensitivity and better electrochemical resolution techniques when compared to CV studies. In this aspect, DPV analysis is chosen in this study for NFT determination on NiFe/*f*-MWCNT/SPCE modified electrode. Figure 7A illustrates NFT response for DPV analysis with different concentrations ranging from 0.1 to 352.4 µM in 0.05 M of PBS (pH = 7.0) at a scan rate of 50 m V s^−1^. It is clearly illustrated that when the concentration of NFT has increased progressively the reduction peak current also increased in increasing order of the concentration from 0.1 to 352.4 µM. Where Fig. 7B illustrates the calibration curve plot for NFT which is derived by using Fig. 7A DPV analysis. Continuing two linear range peaks were observed which is attributed for lower concentration potential and higher concentration potentials (lower = 0.1 to 24.8 µM Higher till 352.4 µM) where two linear regressions are shown in stating I_pc_ = 0.101(NFT) + 0.2567 (µM) with R^2^ = 0.9927 for lower concentration and I_pc_ = 0.4017 (NFT) + 1.978 (µM) with a R^2^ value of 0.9922 (standard error (slope) =  ± 0.001). Simultaneously, the limit of detection (LOD) and limit of quantification is calculated using the following equations^13^, LOD = 3 × SD (analytical curve slope) which is obtained from LOD = 3S_B_/s here, S_B_ = standard deviation of the blank signal obtained, s = sensitivity. By using the above equation LOD is calculated to be 0.03 µM, where sensitivity is of 11.45 µA µM^−1^ cm^−2^. Furthermore, with the results which are obtained from analytical parameters like LOD, linear range and sensitivity by comparing with previous reports Table 1. the proposed sensor shows superior and satisfactory results. As per, best of our knowledge no reports were observed with the proposed NiFe/*f*-MWCNT/SPCE electrode for determination of NFT. These promising results obtained through electrochemical sensing is may be due to the large surface area implying to absorb the analyte more, and iconic conductivity of the composite with excellent synergic effect between NiFe and *f*-MWCNT resulting a host–guest interaction.

**Table 1:** Previous reported electrochemical sensors.

Material	Method	Response time (s)	Linear range (µM)	LOD (µM)	Ref
Nd_2_Mo_3_O_9_	DPV	–	0.1–1,331	13 nM	^10^
Poly(5-amino-2-mercapto-1,3,4-thiadiazole)	DPV	–	2–25 mg L^−1^	0.65 mg L^−1^	^11^
Boron-doped diamond	SWV	–	0.497–5.66	8.18 nM	^12^
Cetrimide	CV	–	0.1–20	0.06 ng mL^−1^	^13^
β-CD/CNF	i-t	5	0.004–308	1.8 nM	^47^
MgFe_2_O_4_	DPV	8	0–342.6	33 nM	^48^
NiFe/*f*-MWCNT	DPV	3	0.1–352.4	0.03 µM	This work

### Interference, stability, and reproducibility

**Figure 8:**
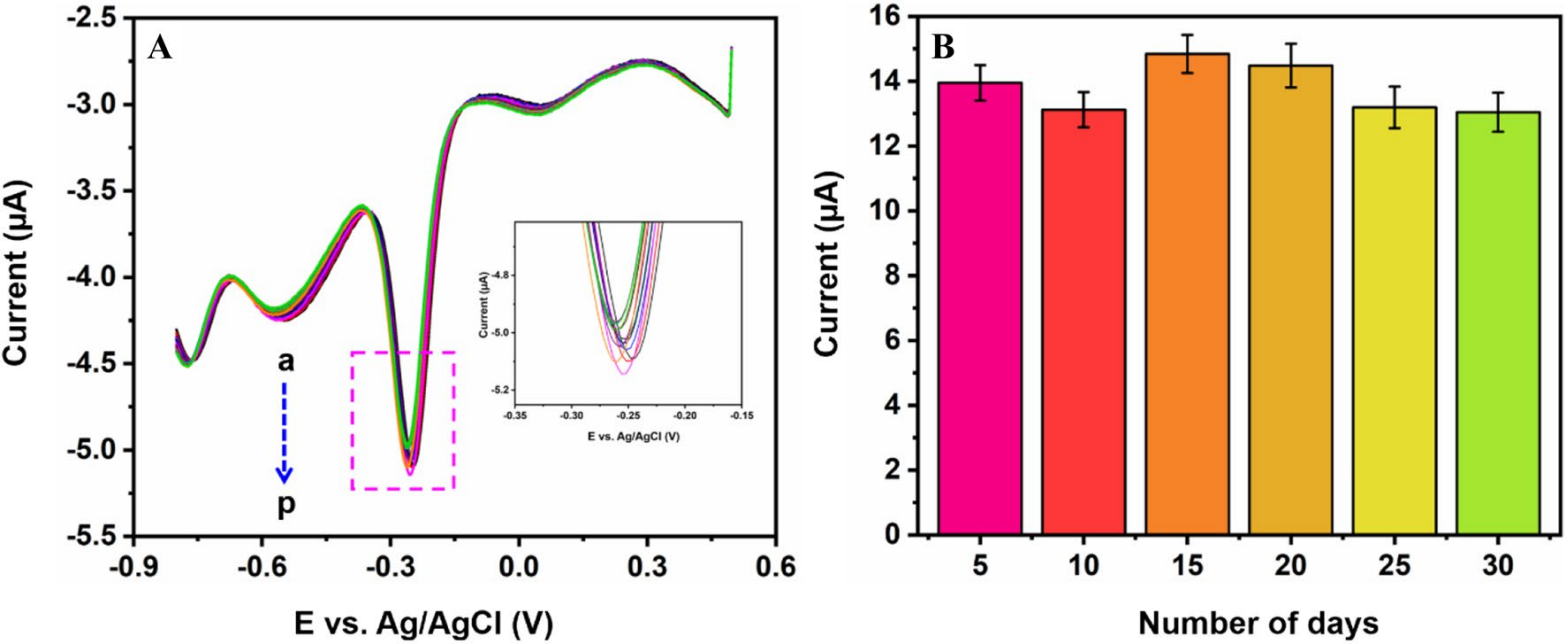
(**A**) DPV response for NFT in the presence of different interferents (a to p), (**B**) stability of modified electrode concerning total no. days vs current response acquired for NFT.

In assessing any new sensor selectivity, stability and reproducibility are considered as major parameters. To examine the overall possibilities of selectivity which interferes are added to DPV NFT assay in pH 7.0 (0.05 PBS). In studying the selectivity different interfering compounds are used like antibiotics, anti-cancer drugs, environmental pollutants and biological electroactive samples such as (a) flutamide (Flu), (b) dopamine (DA), (c) uric acid (UA), (d) ascorbic acid (AA), (e) glucose (Glu), (f) caffeic acid (CA), (g) catechol (CC), (h) metronidazole (MTZ), (i) hydrogen peroxide, (HP), (j) paracetamol (PCT), (k) vitamin B6, (l) vitamin B9, (m) 4-Nitrophenol (4-NP), (n) 4-Nitrobenzene (4-NBZ), (o) 4-aminophenol (4-AP), and (p) Chloramphenicol (CAP) DPV responses is illustrated in Fig. 8A in Inset clear illustration of interfering compounds is displayed. Although, the reduction peak current of NFT has not implied any effect on the interfering compounds. From the inset image, it is seen that a tenfold excess concentration peak variation is observed for biological compounds like Flu, DA, UA, AA, Glu, CA, and catechol CC, although only fivefold excess is observed for rest of the environmental pollutants and drug samples^43^. Subsequently, the interfering response is not more than 5% for a reduction peak signal. Thus, it suggests that NiFe/*f*-MWCNT/SPCE electrode has displayed quite excellent anti-interference ability which enlarges the scope for effective detection of real sample analysis in NFT for human serum and urine sample analysis.

To analyze the stability conditions of the NiFe/*f*-MWCNT/SPCE electrode is evaluated with DPV analysis in pH 7.0 (0.05 M PBS). Figure 8B illustrates the DPV signals obtained by measuring 50 µM of NFT which is stored at room temperature. The results disclose that a reduction peak current response is only 3% even after a week which continued till one month, stating good storage capability of the proposed sensor^44,45^. Continuing, to the proposed sensor NiFe/*f*-MWCNT is checked with 8 multiple measurements using a similar electrode for better repeatability enhancement analysis. In which 50 µM of NFT existed with 0.05 M of PBS (pH 7.0) to which relative standard deviation is 3.12%.

### Determination of NFT in real sample analysis

Real sample analysis is carried out to know the practical feasibility of NiFe/*f*-MWCNT modified sensor, where the composite is analyzed in bio-fluids like human serum and urine using the standard addition method. Before using for standard deviation urine samples are centrifuged at 10,000 rpm for 5 min obtained supernatant is collected and used further^22,46^. Primarily, 1 mL of urine and human serum are mixed with 9 mL of 0.05 PBS in pH 7.0 thorough shake was performed to mix it. DPV analysis is used for real sample analysis to NiFe/*f*-MWCNT/SPCE electrode and electrochemical analysis was performed to know the peak current response in respect of pH analysis and is quantified accordingly. Obtained results are given in Table 2. This proves that the recovery of human serum and urine samples is merely about 99.6% with an RSD value of 1.03 and 1.27% (n = 3). Thus, a promising NiFe/*f*-MWCNT electrode is constructed as it has exhibited satisfying results in reliability and accuracy for NFT detection.

**Table 2:** Detection of NFT with various biological samples using the NiFe/*f*-MWCNT electrode.

Sample	Added (µM)*	Detected (µM)^a^		Detection rate (%) (Mean ± RSD) (n = 3)^b^
		DPV	HPLC	
Human serum	5	4.96	4.92	99.2 ± 0.014
	10	9.92	9.91	99.2 ± 0.019
	20	19.96	20.03	99.8 ± 0.017
	30	29.94	29.08	99.8 ± 0.021
Urine	5	4.91	4.94	98.2 ± 0.012
	10	9.97	9.94	99.7 ± 0.023
	20	19.94	19.99	99.7 ± 0.011
	30	29.95	30.01	99.8 ± 0.015

*standard addition.

^a^Average value from the sum of three measurements.

^b^Relative standard deviation (RSD) of (n = 3 is the sum of three individual measurements).

In brief, a facile, cost-effective and rapid response electrochemical sensor based on NiFe nanospheres anchored on *f*-MWCNT is been reported in NFT detection. Whereas the results obtained from electrochemical sensing proves the developed sensor has the excellent catalytic ability, analytical performance with a wide dynamic range of 0.1 to 352.4 µM and with a very low detection of 0.03 µM. Due to low impedance, NiFe nanospheres when anchored on *f*-MWCNT delivered synergic effect which enriched active sites for NFT diffusion leading in achieving a good sensing performance. Continuing, it also exhibited good selectivity for NFT in the presence of co-existing interferents leading to analyze real samples like human serum and urine samples which have given satisfactory recoveries in practicality. Thus, offering a new idea for synthesizing of nanostructured bimetallic carbon sourced catalyst and its electrocatalytic sensing.

## Experimental section

### Materials

Nickel nitrate hexahydrate (Ni(NO_3_)_2_·6H_2_O), iron nitrate nonahydrate (Fe(NO_3_)_3_·9H_2_O), Multiwall—Carbon Nanotubes (MWCNT), Nitrofurantoin (NFT), ethylene glycol (HOCH_2_CH_2_OH), disodium hydrogen phosphate (Na_2_HPO_4_), monosodium dihydrogen phosphate (NaH_2_PO_4_), ethanol, acetone are purchased from Sigma-Aldrich of analytical grade with a purity of ~ 99% and used as received. Different pH values for phosphate buffer solution were prepared by NaH_2_PO_4_·H_2_O and Na_2_HPO_4_·2H_2_O by dissolving in double-distilled water (ddH_2_O).

### Characterization

X-ray diffraction (XRD) analysis is persuaded by using an XPERT-PROF diffractometer (PAN-alytical B.V., Netherlands) with Cu-Kα radiation of λ = 1.5406 Å. Surface morphology with elemental mapping is conducted using Transmission electron microscopy (TEM) supported with TECNAI G2 microscope in range of 40–200 kV and Scanning transmission electron microscopy-energy dispersive X-ray analysis (STEM-EDX). X-ray photoelectron microscopy (XPS) studies were carried out using thermo scientific multilab 2000 for investigating the electronic state analysis of Ni, Fe, and C in the prepared sample. Absolute pure pH meter (pH500) attached with a pH glass electrode is used for pH studies. The electrochemical analysis is carried out in CHI 900 workstation. A conventional three-electrode system is been engaged, with the as per prepared screen-printed carbon electrode (SPCE) as a working electrode (surface area of electrode = 0.035), saturated Ag/AgCl performed as reference electrode and platinum wire deprived as a counter electrode.

### Synthesis of NiFe nanospheres

The NiFe nanospheres are prepared in a simple hydrothermal procedure. In a typical synthesis procedure, 0.01 M of nickel acetate and 0.01 M of iron acetate are mixed with ethylene glycol and distilled water (1:2) in a mixture of 80 mL solution under vigorous stirring at room temperature. Continuing the mixture is probe sonicated for 30 min, later the solution is transferred into a 100 mL Teflon-lined autoclave which is maintained at 160 °C for 16 h. further, the sample is removed after cooling and is washed thoroughly multiple times with distilled water and ethanol followed by centrifugation at a speed of 4,500 rpm for 30 min each time. The obtained mixture is dried out by using a hot air oven at 80 °C overnight. MWCNTs are functionalized (Synthesis procedure S1) and are mixed with as-prepared NiFe nanospheres by adding the desired amount with 5 mL of distilled water and ultrasonicated for 1 h, further the mixture is dried in hot air oven to attain NiFe-*f*-MWCNT hybrid composite. Finally, the attained NiFe-*f*-MWCNT hybrid composite is synthesized and used in the further process for characteristically and electrochemical analysis.

### Fabrication of NiFe-*f*-MWCNT on the SPCE electrode

A disposable screen-printed carbon paste electrode (SPCE) is purchased from Zensor-Labs (Taiwan). Where, the electrode modification process is persuaded by coating approximately 6 µL of NiFe-*f*-MWCNT on the active surface of the SPCE, later dried at 600 °C in a vacuum air oven for 15 min. Only modified SPCE electrodes are used completely in the electrochemical analysis study when SPCE is not used it is stored under 4 °C in a PB solution (pH 7).

## Supplementary information

Supplementary information
