# Supplementary material for: Nano assembly of NiFe spheres anchored on f-MWCNT for electrocatalytic reduction and sensing of nitrofurantoin in biological samples
Source: Sci Rep. 2020 Jul 23;10:12256. doi: 10.1038/s41598-020-69125-5 (PMC7378214; doi:10.1038/s41598-020-69125-5)
Supplement: Supplementary file 1 — Supplementary information [file 41598_2020_69125_MOESM1_ESM.docx]

**Nano assembly of NiFe spheres anchored on *f*-MWCNT for electrocatalytic reduction and sensing of Nitrofurantoin in biological samples**

**Kuo-Yuan Hwa*^a,b,c^, Tata Sanjay Kanna Sharma ^a,b^**

^a^Graduate Institute of Organic and Polymeric Materials, National Taipei University of Technology, Taipei, Taiwan (R.O.C).

^b^Department of Molecular Science and Engineering, National Taipei University of Technology, Taipei, Taiwan (R.O.C).

^c^Center for Biomedical Industry, National Taipei University of Technology, Taipei, Taiwan (R.O.C).

**Corresponding author**

Kuo-Yuan Hwa* Email: kyhwa@ntut.edu.tw

Phone number: 02-27712171 ext.2419 (0), 2439, 2442 (lab).

**S.1. Functionalization of MWCNT**

MWCNT was purchased for thermo Scientifics, obtained MWCNT is dispersed in a combination mixture of nitric acid of about 0.6 ml and double-distilled water of about 100 ml. later, acquired solution is transferred in to a round bottomed flask continued vigorous magnetic stirring for 12 hours at 40^0^C temperature. Therefore, the mixture is refluxed for 18 hours at 800C temperature. Further oxidized MWCNT was washed several time using ethanol and double-distilled water to remove the unreacted particles. Obtained liquid *f*-MWCNT mixture is filtered with 0.2 µM pore sized Whatman filter paper using high-pressure suction pump for about 1 hour. pH was adjusted and achieved after several times of washing eliminating the acid concentration. Afterwards, the obtained product was kept in freeze drier in a -60^0^C for 48 hours for drying. To which obtained mixture is transferred to Nano pulverizer (THINKY NP-100) for 5 mins to acquire a fine *f*-MWCNT.


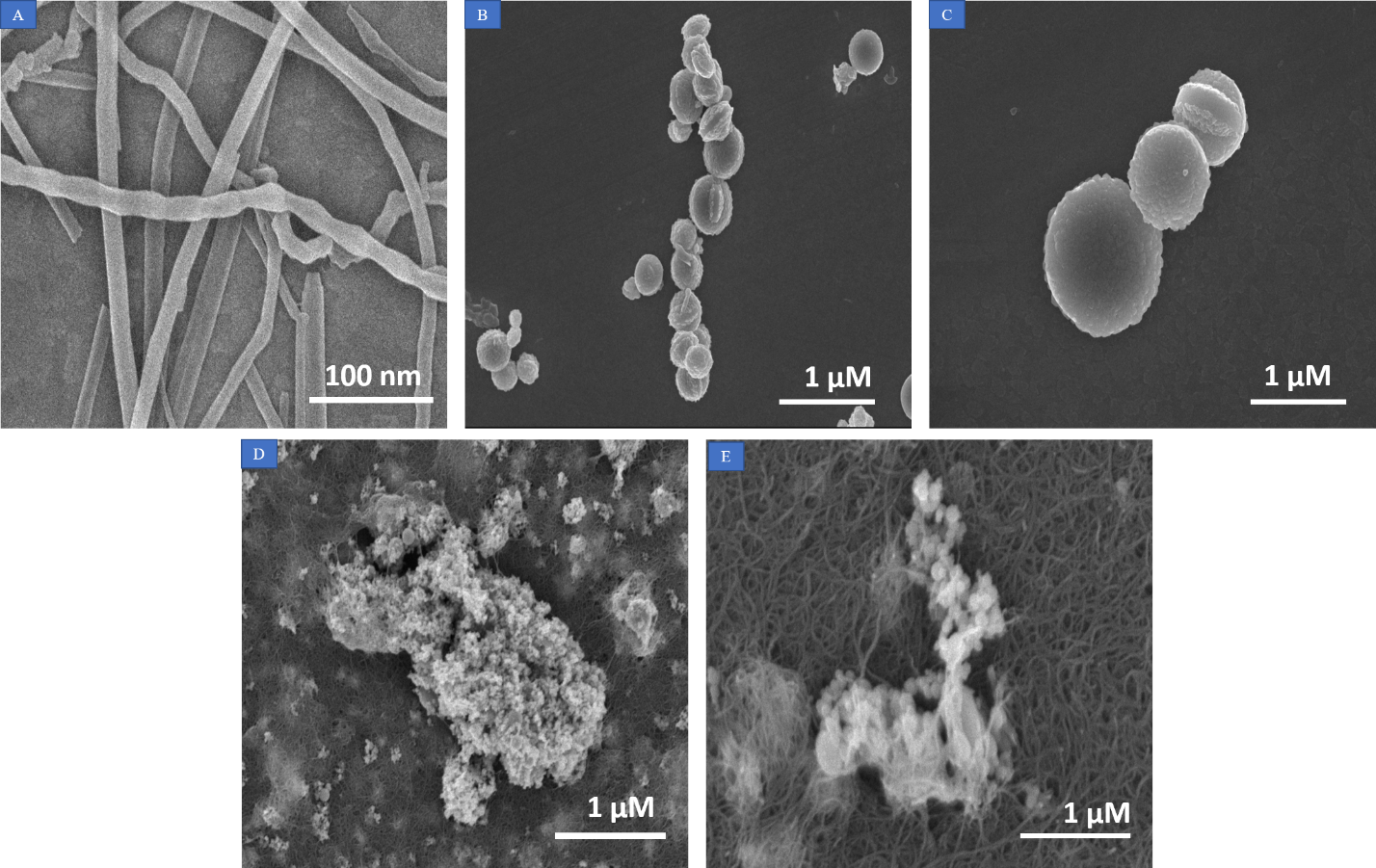


**Fig.S.2.** FE-SEM images of NiFe-*f*-MWCNT composite **(A)** *f*-MWCNT **(B-C)** NiFe and **(D-E)** NiFe-*f*-MWCNT.


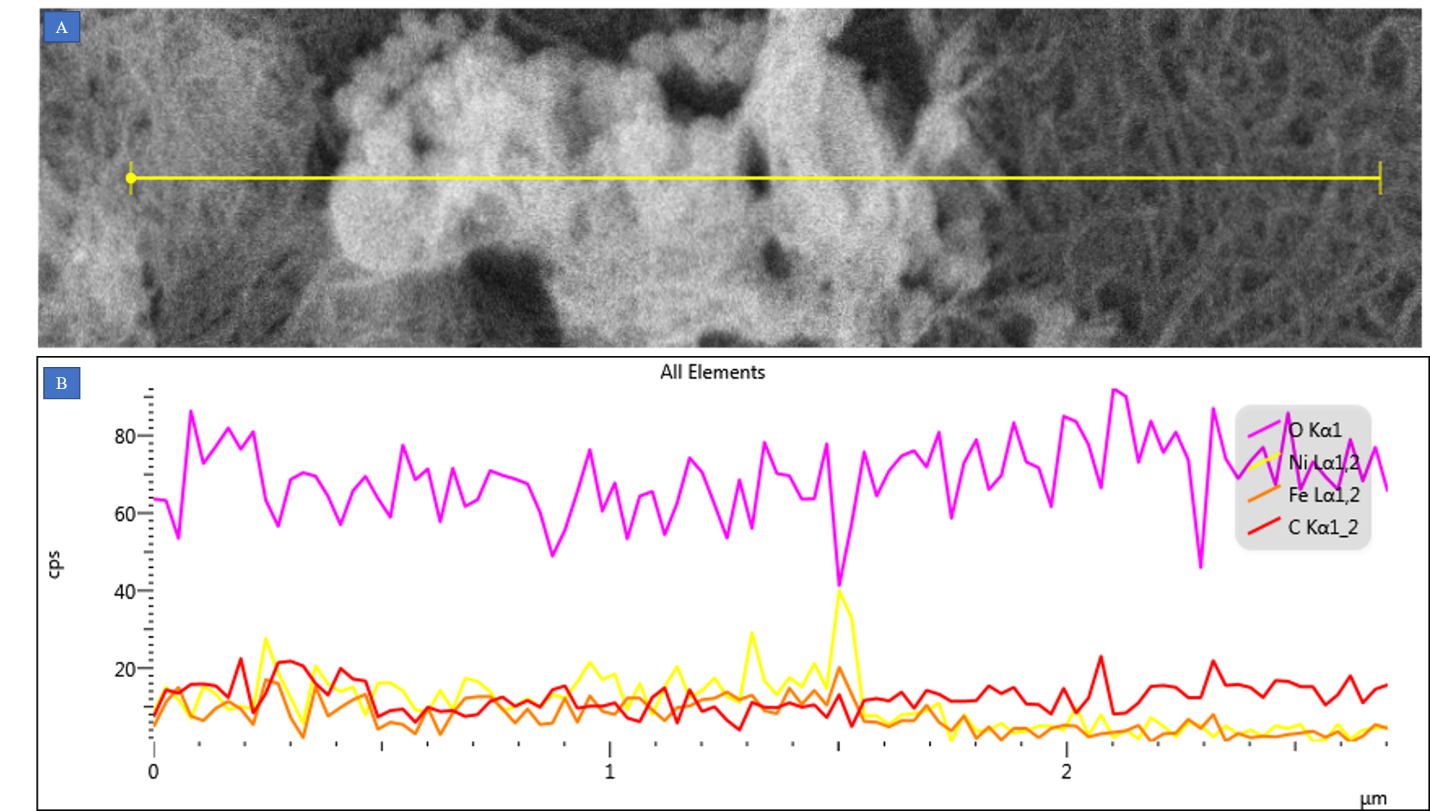


**Fig.S.3.** Line mapping of NiFe/*f*-MWCNT composite.


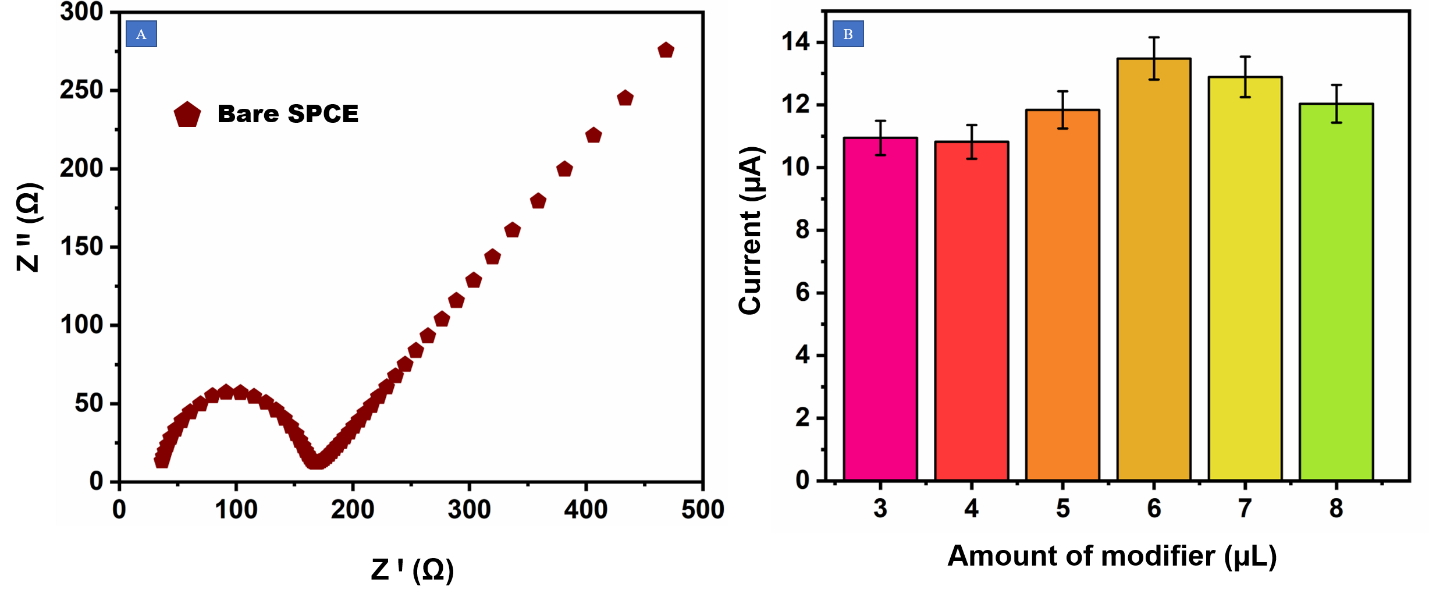


**Fig.S.4.** **(A)** EIS spectra of bare SPCE. **(B)** Effect of drop-casting of NiFe/*f*-MWCNT composite peak current response for 200 µM of NFT.
